# Therapeutic Effectiveness of Anticancer Phytochemicals on Cancer Stem Cells

**DOI:** 10.3390/toxins8070199

**Published:** 2016-06-30

**Authors:** Jisun Oh, Lynn Hlatky, Yong-Seob Jeong, Dohoon Kim

**Affiliations:** 1 School of Food Science and Biotechnology (BK21 Plus), Kyungpook National University, Daegu 41566, Korea; 2 Center of Cancer Systems Biology, Tufts University School of Medicine, Boston, MA 02135, USA; hlatky@cancer-systems-biology.org; 3 Department of Food Science and Technology, Chonbuk National University, Jeonju 54896, Korea; ysjeong@jbnu.ac.kr; 4 Department of Integrative Physiology and Pathobiology, Tufts University School of Medicine, Boston, MA 02111, USA

**Keywords:** cancer, cancer stem cells, anticancer, phytochemicals, polyphenols

## Abstract

Understanding how to target cancer stem cells (CSCs) may provide helpful insights for the development of therapeutic or preventive strategies against cancers. Dietary phytochemicals with anticancer properties are promising candidates and have selective impact on CSCs. This review summarizes the influence of phytochemicals on heterogeneous cancer cell populations as well as on specific targeting of CSCs.

## 1. Introduction

While cancer cells are heterogeneous in their tumorigenic potential, a small subset of tumor cells—cancer stem cells (CSCs)—have uniquely high potency for initiating tumorigenesis. These CSCs are postulated to proliferate with unlimited potential, exhibit high resistance to therapy, and have the ability to fuel tumor regrowth post-treatment. Considering the potential of CSCs in both the initial development of cancer and in post-treatment regrowth, they have become a critical focus for the development of new therapeutic strategies. Numerous studies have demonstrated that phytochemicals that have antioxidative properties have anticancer effects. This review summarizes the influence of phytochemicals on cancer cell populations, highlighting the importance of those known to selectively target CSCs and discussing their mechanisms of action.

## 2. Cancer Stem Cells

Cancer cells within a tumor consist of various clonal subpopulations, thereby exhibiting heterogeneity across many properties, such as genetic variations, marker expression, and proliferative and metastatic potential, and sensitivity to drugs [1]. Considering this heterogeneity of cancer cells, two models have been proposed regarding the origin of tumorigenesis: (i) assembly of diverse cancer clones (referred to as the “stochastic model”) and (ii) generation of multiple subclones from a single clone (referred to as the “hierarchical model”) [2,3]. In the stochastic model, most cancer cells are capable of proliferating extensively and forming new tumors in cooperation with intrinsic and extrinsic factors. According to this model, tumorigenesis occurs randomly from somatic cells undergoing transformation. In the hierarchical model, on the other hand, only a distinct subpopulation of cancer cells, CSCs, has the ability to extensively proliferate and initiate tumor formation and growth. According to this model, tumorigenesis originates from the CSCs which can be enriched based on unique cellular features [4]. This means only the CSCs possess the cellular capacity to replenish the tumor population. It has recently been shown that cancer non-stem and cancer stem cells may plastically interconvert under particular conditions [5]. However, this does not diminish the fact that the achievement of CSC status, either naturally or by cellular plasticity, is necessary and sufficient for tumorigenicity.

Current treatment approaches in cancer are grounded in the need to kill the majority of cancer cells, based on the stochastic model. However, in many instances where such efforts have not been successful in the treatment of solid cancers, it may be time to refocus our thinking around the hierarchy model in trying to explain resistance to anticancer therapeutics and tumor recurrence [6].

### 2.1. Cancer Stem Cell Hypothesis

The hierarchical model for tumorigenesis maintains that CSCs are the origin of tumor formation, metastasis, and relapse. In the past two decades, conclusive evidence has demonstrated the existence of CSCs. In 1997, Bonnet and Dick reported a subset of cells—leukemic stem cells—that was isolated from the blood of acute myeloid leukemia, originating from normal hematopoietic stem cells and capable of self-renewing and differentiating into leukemic blasts in immunocompromised mice [7]. This study suggested that the hematopoietic stem cells may be susceptible to leukemic transformation and progression, and was presumably responsible for the hierarchical organization of the leukemic clone. Subsequently, CSCs from solid human tumors, such as breast cancer [8,9], prostate cancer [10] and brain tumors [11], were also isolated and identified on the basis of their tumorigenic capability and cell surface antigen expression. With accumulating evidence for the existence of CSCs within a myriad of other solid tumors [12], the CSC hypothesis has been strongly considered to be a fundamental underpinning of cancer biology that should be considered in thinking about the development of effective cancer therapeutic strategies.

### 2.2. Cellular Properties of Cancer Stem Cells

Like normal tissue stem cells, CSCs are capable of self-renewal and differentiation into cancer progenitors or mature cancer cells. CSCs can repopulate clonally by cell division (symmetric or asymmetric) or uncontrolled proliferation [13]. Thus, it is thought that CSCs may derive either from normal stem cells that undergo genetic or epigenetic alterations, or from cancer cells (not fully differentiated; cancer progenitor cells) that acquire the potential for unrestrained proliferation [3,14,15]. Although the exact cellular origin of CSCs may be a critical issue in cancer research, it is still unresolved.

In terms of CSC phenotypes, CSCs can be recognized by specific antigens that are expressed, or not, on the cell surface (Table 1). The antibodies against these antigens are generally used for phenotypic characterization or prospective isolation of CSCs (reviewed in [16]). Thus, the specific antigens are considered molecular markers for the validation of CSCs. However, one needs to be cautious in defining the cells expressing these markers as CSCs, since none of these markers are exclusively made by CSCs. Furthermore, CSC phenotypes, even from the same tumor, can exhibit different markers owing to the possible presence of multiple CSC pools, technical variations, or the occurrence of epigenetic alterations [6]. Thus, a combination of different molecular markers, together with epigenetic profiling, may refine the identification of the CSC phenotype.

Furthermore, tumor tissues composed of malignant cells, including CSCs, reside in the perivascular niche, which is the milieu that nourishes cancer cells and consists of vasculature, hematopoietic cells, inflammatory cells, and myofibroblasts. Although the niche is not indispensable for the sustainment of all types of cancer, mutual interactions between CSCs and the microenvironment are known to profoundly influence cellular properties, such as cell fate and secretory profiles, for certain types of cancer cells [36,37,38].

## 3. Anticancer Phytochemicals Targeting CSCs

CSCs are believed to be responsible for the initial formation and growth of cancer, as well as the relapse of cancer after treatment, due to the fact that CSCs are more resistant to conventional therapeutic treatment than differentiated cancer cells [39]. Thus, it would have important implications for cancer prevention and further therapy if treatment could specifically target CSCs while avoiding damage to normal stem cells. Based on their unique features [40,41] and dynamics [42] (reviewed in [6]), CSCs can be targeted by several strategies, such as inhibition of self-renewal, induction of differentiation into mature cancer cells, and sensitization to anticancer agents.

**Figure 1:**
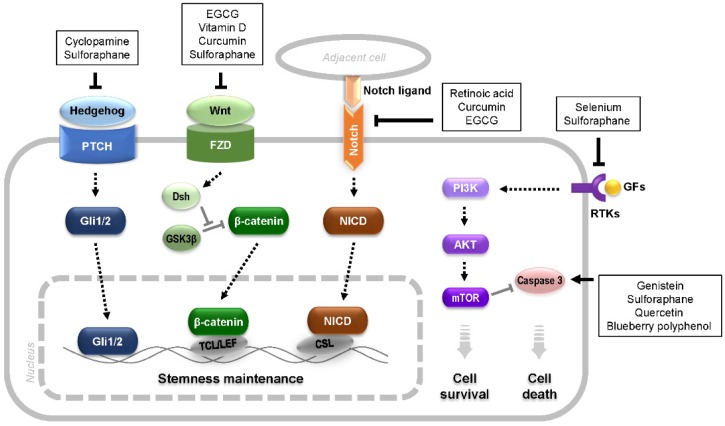
Selected phytochemicals that target signaling pathways involved in stemness maintenance and survival of CSCs. PTCH: Patched, receptors for hedgehog; FZD: Frizzled, receptors for Wnt; Dsh: disheveled, a downstream molecule of FZD; NICD: Notch intracellular domain, a Notch fragment cleaved by γ-secretase; RTKs: receptor tyrosine kinases; GFs: growth factors.

Among approved anticancer drugs, approximately 50% are either natural products or their derivatives [43], primarily from plants, microorganisms, and seeds [44]. Numerous plants have been reported to have anticancer effects [45] or to complement conventional therapeutics by targeting various hallmarks of cancer [46]. Plant-derived natural chemicals, termed phytochemicals, that are used for cancer treatment and that target CSCs are addressed in this section (Figure 1).

### 3.1. Anticancer Phytochemicals

A large number of phytochemicals, i.e., chemical compounds produced from plants, including vegetables, fruits, and grains, have been reported to possess anticancer properties and are promoted for cancer prevention and treatment [44,45,47,48,49] (Table 2). Phytochemicals have been shown to interfere with stabilization of the microtubule structure, thereby inhibiting mitosis and cancer cell propagation. Vincristine and vinblastine, isolated from the leaves of Madagascar periwinkle, were the first phytochemicals to be used clinically in combination with other anticancer agents in lymphomas, leukemias, and breast and lung cancers. Paclitaxel (Taxol), which was originally discovered in the bark of the Pacific yew tree, is one of the most effective and widely used phytochemical compound against breast and ovarian cancers [50,51].

Another group of phytochemicals, known as polyphenols, has been shown to have free-radical scavenging activity, working like antioxidants. Epigallocatechin-3-gallate (EGCG), a polyphenol from the leaves of *Camellia sinensis* (processed to green tea), has been used effectively against breast cancer [47]. EGCG was demonstrated to limit cancer cell proliferation by reducing DNA methylation through the inhibition of DNA methyltransferase together with reactivation of the silenced tumor suppressor genes. Curcumin (diferuloylmethane), a polyphenol isolated from the rhizome of the turmeric plant, has also shown therapeutic efficacy on numerous disorders, including cancer [52]. Curcumin is reported to inhibit NF-κB signaling that triggers the intracellular inflammatory response as well as cell-cycle-associated genes. By arresting the cell cycle and inducing apoptosis through the relaying pathways, curcumin interferes with angiogenesis and reduces tumor invasion.

An additional group of anticancer phytochemicals functions as inhibitors of topoisomerase I or II, which are the nuclear enzymes that control DNA supercoiling, eliminate tangles in the chromatin structure, and allow DNA to be replicated and transcribed. Thus, topoisomerase inhibitors can act as anticancer agents by inducing a delay of the cell cycle, followed by cell death [44]. β-Lapachone from the bark of the lapacho plant [53], camptothecin from the bark/stem of *Camptotheca* (the Chinese happy tree), and podophyllotoxin from the root of the Mayapple plant are examples of phytochemicals inhibiting topoisomerases in cancer cells [54,55].

### 3.2. Phytochemicals Targeting CSCs

Several phytochemicals have been reported to intervene in signaling pathways critical for stemness maintenance of CSCs or to modulate the CSC phenotype [56,57]. The hedgehog, Wnt/β-catenin, and Notch-mediated signaling pathways play important roles in CSC self-renewal and differentiation [58]. Considering that tumorigenesis might be derived from CSCs in which these pathways are aberrantly regulated, the signaling molecules in these pathways may be of particular interest for targeting CSCs [59]. Multiple studies have demonstrated that cancer cell growth can be suppressed by specific inhibitors of these pathways [60,61]. Specific phytochemicals have been reported to influence these signaling pathways. Cyclopamine, initially found in the corn lily (*Veratrum californicum*), targets hedgehog signaling [62,63,64,65]. EGCG inhibits Wnt/β-catenin signaling, which affects the self-renewal and invasive abilities of certain CSCs [66,67,68]. In addition, retinoic acid, the active molecule derived from vitamin A in animals, has been demonstrated to differentiate CSCs or deplete their formation in glioblastoma by downregulating Notch signaling [69,70]. Vitamin D or its analogs can inhibit Notch and/or Wnt/β-catenin signaling and thus induce CSC differentiation [71,72]. Furthermore, curcumin from turmeric and piperine from black and long peppers, well-known anticancer phytochemicals, have also been shown to target breast CSCs by inhibiting Notch and/or Wnt/β-catenin signaling [73]. However, it should be recognized that the inhibition of these self-renewal pathways can affect normal stem cell function as well.

Akt/mTOR signaling is known to be critical for CSC survival and invasion. Akt inhibition causes a preferential induction of apoptosis and reduction of CSC motility [57]. Selenium, as an anticarcinogenic nutrient [74], functions biologically in a form of selenoproteins that are oxidoreductase scavenging oxidants [75]. It was shown that selenium involvement in the modulation of arachidonic acid metabolism could trigger apoptosis of leukemia CSCs [76], and that the apoptosis was regulated through Akt/mTOR signaling [77,78]. However, another study indicated that the biological benefits of selenium supplementation may not necessarily be due to its activity of lowering the level of reactive species [79]. Thus, the exact mechanisms underlying selenium-mediated CSC apoptosis awaits further elucidation. In addition, sulforaphane from cruciferous vegetables, such as broccoli, has been shown to reduce breast and pancreatic CSC viability by affecting Wnt/β-catenin signaling [80,81] or hedgehog signaling [82,83]. Several studies have also demonstrated that sulforaphane can downregulate Akt signaling in various solid cancers [84,85] and breast CSCs [86].

As described above, the polyphenols EGCG and curcumin are known to exert their anticancer effects though antioxidative activity. Polyphenols can inhibit proliferation and/or induce caspase-3-dependent apoptosis of cancer cells via the above-mentioned vital signaling pathways or their cross-talk [87,88]. Being ubiquitously present in nature, polyphenols can be richly extracted from a broad range of fruits, grains, and vegetables, and include flavonoids (categorized into flavones, isoflavones, catechins, and anthocyanins) and lignans. Several studies have suggested that phenolic compounds or polyphenol-containing extracts can influence CSCs as well as cancer cells [89]. Montales et al. reported that the soy isoflavone genistein or blueberry polyphenol treatment could reduce the population of breast CSC-like cells in vitro [90]. Appari et al. showed that a mixture of green tea catechins in combination with sulforaphane and quercetin remarkably inhibited the viability and migration and induced apoptosis of pancreatic CSCs [91]. Lu et al. showed that anthocyanins (i.e., phenolic compounds found in grapes, eggplants, red cabbages, and radishes) can inhibit cancer invasion and epithelial-mesenchymal transition of uterine cervical cancer cells [92]. Quercetin, a flavonol that can be enriched from apples, onions, teas, and berries, has demonstrated efficacy against pancreatic and head/neck CSCs [93,94]. The synergistic effect of quercetin with EGCG or sulforaphane in eliminating prostate or pancreatic CSCs has also been described [95,96]. Thus, polyphenols, including flavonoids, may be considered promising anticancer agents for targeting various CSCs, although further extensive investigation on their bioavailability and working mechanisms at the cellular and molecular levels have yet to be done.

On the basis of the accumulating evidence supporting the anticancer activity of phytochemicals, there have been some clinical studies. Recent clinical trials have shown the effectiveness of curcumin, green tea catechins, including EGCG, and sulforaphane against various cancers (reviewed in [97,98]). In particular, curcumin showed its therapeutic potency in human clinical trials [99] via targeting CSCs [100]. However, the low bioavailability of curcumin makes its therapeutic use challenging. To overcome this issue, several strategies such as structural modifications or special formulations are being tried. It is expected that in vitro and in vivo functional studies using the compounds or their derivatives targeting CSCs may provide useful information on eliminating CSCs and inhibiting tumorigenesis.

Along with the emerging evidence supporting the beneficial effects against CSCs as well as cancer cells, the beauty of phytochemicals is the fact that they are naturally present in edible plant materials. This warrants the assurance of safety for ingestion. In addition, certain phytochemicals sensitize CSCs to conventional chemotherapeutic agents by interfering with the key signaling pathways for cell survival, stemness maintenance of CSCs, or both. Thus, synergistic effects are expected when the CSC-targeting phytochemicals and chemotherapeutic drugs are used combinatorially [98]. However, rigorous examinations are required to test the potential adverse effects, such as the counteraction of phytochemicals against chemotherapeutic drugs and the additive toxicity of phytochemicals [101].

## 4. Summary and Conclusions

Tumors comprise of phenotypically and functionally heterogeneous cells. Based on this feature, two models have been established regarding tumorigenesis: the stochastic model and the hierarchical model. The latter model postulates a hierarchical organization of diverse populations of cells and premises the presence of CSCs that account for the sustainment of tumorigenesis. Thus, control of CSCs may be a necessary first step in an effective strategy for cancer treatment. Dietary phytochemicals can exert influence at all stages of cancer development. Since some of the phytochemicals are already known to affect CSC viability and fate, extensive and intensive studies of these compounds should provide insight into their pharmaceutical efficacy for cancer prevention and therapy.

## Figures and Tables

**Table 1:** Phenotypic markers for cancer stem cell identification in various tissues.

Tumor	CSC Marker	References
Leukemia	CD34^+^/CD38^−^	[7]
Breast	CD24^−^/CD44^+^/Lineage^−^/ALDH1^+^	[8,9]
Prostate	CD44^+^/CD133^+^/Integrin α2β1^high^	[10,17,18]
Brain	CD133^+^	[11]
Stomach	CD44^+^/CD133^+^	[19,20,21,22]
Pancreas	CD24^+^/CD44^+^/CD133^+^/ESA^+^	[23,24,25]
Colon	CD44^+^/CD133^+^/ALDH1^+^	[26,27]
Ovary	CD133^+^/ALDH1^+^	[28,29]
Lung	CD133^+^	[30,31,32]
Liver	CD90^+^	[33,34,35]

CSC: cancer stem cell; CD24: heat stable antigen; CD34: hematopoietic progenitor cell antigen; CD38: cyclic ADP ribose hydrolase; CD44: hyaluronate receptor; CD90: Thy-1; CD133: prominin-1; ALDH1: aldehyde dehydrogenase 1A1; ESA: epithelial surface antigen.

**Table 2:** Examples of plant-derived anticancer phytochemicals.

Function	Phytochemicals	Plant Derived from
Interference of microtubule stabilization	Vincristine, vinblastine	Madagascar periwinkle
	Paclitaxel	Pacific yew tree
Limitation of cell proliferation	Epigallocatechin-3-gallate	*Camellia sinensis*
	Curcumin	Turmeric
Disruption of chromatin structure	β-lapachone	Lapacho plant
	camptothecin	Camptotheca
	podophyllotoxin	Mayapple plant
